# Comparison of the burden of musculoskeletal disorders between China and worldwide data using the global burden of disease dataset from 1990 to 2021

**DOI:** 10.1080/07853890.2025.2529578

**Published:** 2025-07-13

**Authors:** Zhongbiao Nie, Ran Zhang, Yaoyao Guo, Xiang Wang, Kai Zhang, Haoliang Zhao

**Affiliations:** a Department of Pharmaceutical, Shanxi Bethune Hospital, Shanxi Academy of Medical Sciences, Tongji Shanxi Hospital, Third Hospital of Shanxi Medical University, Taiyuan, China; b Nephrology Department, Affiliated Hospital of Shanxi University of Chinese Medicine, Taiyuan, China; c Department of Pain, Shanxi Bethune Hospital, Shanxi Academy of Medical Sciences, Tongji Shanxi Hospital, Third Hospital of Shanxi Medical University, Taiyuan, China; d Department of Orthopaedics, Shanxi Bethune Hospital, Shanxi Academy of Medical Sciences, Tongji Shanxi Hospital, Third Hospital of Shanxi Medical University, Taiyuan, China

**Keywords:** Musculoskeletal disorders, incidence, prevalence, mortality, DALYs

## Abstract

**Objectives:**

This study was to compare the worldwide burden of musculoskeletal (MSK) disorders with the age and gender-specific trends of MSK disorders in China and globally between 1990 and 2021.

**Methods:**

Using publicly available data from the Global Burden of Disease (GBD) database from 1990 to 2021. Examined the features of the burden of MSK disorders in China and globally, including age and gender-specific trends in incidence, prevalence, mortality, disability-adjusted life years (DALYs), and related age-standardized measures of MSK disorders. To represent the trends in the burden of MSK disorders, the average annual percentage change (AAPC) was computed using Joinpoint. Age, gender were important parameters that were used a comparative study of the disparities in the burden of MSK disorders between China and the global.

**Results:**

The worldwide ASIR of MSK disorders rose from 4641.50 to 4358.54 between 1990 and 2021, and China fell from 4039.13 to 3634.09 per 100,000. The worldwide ASPR rose from 19178.47 to 19836.76, China, went from 16966.24 to 17358.70 per 100,000. The ASMR in China declined from 1.22 to 1.10, while the worldwide decreased from 1.55 to 1.47 per 100,000. The ASDR in China declined from 1615.73 to 1578.71, while the worldwide ASDR decreased from 1886.22 to 1916.21 per 100,000. The worldwide AAPC of ASIR, ASPR, ASMR, and ASDR was −0.21%, 0.11%, −0.25%, and 0.04%, respectively. China was −0.34%, 0.09%, −0.41%, and −0.07%, respectively. The burden of MSK disorders was influenced similarly by age and gender.

**Conclusion:**

The burden of MSK disorders rose in China and globally between 1990 and 2021, and it varies with age. Women are more prone than men to acquire MSK disorders. Because of its vast and aging population, MSK disorders continue to be a major public health concern in China and globally.

## Introduction

A variety of disorders affecting the motor system, including muscles, bones, joints, and tendons, are known as musculoskeletal (MSK) disorders. Rheumatoid arthritis (RA), osteoarthritis (OA), neck pain (NP), low back pain (LBP), and gout are common forms of these conditions [1,2]. Joint discomfort, stiffness, and reduced mobility are characteristics of these disorders that can lead to physical disability, depressive symptoms, and other chronic health problems including cardiovascular disease [2,3]. In 2021, MSK disorders were the leading cause of disability worldwide, accounting for the highest number of years lived with disability (YLDs) and the sixth-highest number of disability-adjusted living years (DALYs) [4]. Furthermore, MSK disorders frequently have a chronic and progressive character, which significantly strains financial resources and healthcare systems [2].

According to recent studies, in addition to having the greatest growth in age-standardized prevalence and DALYs rates over the past ten years across all quintiles of Socio-demographic Index (SDI), nations with greater SDI also experienced the only increase in age-standardized incidence rates [5]. A number of environmental and socioeconomic variables interact with the mortality rate of MSK disorders. Countries affected by MSK disorders must systematically implement evidence-based preventative and treatment strategies to reduce the mortality rate linked to these ailments [6]. They should also carefully compare the cost-effectiveness and efficacy of various approaches to reduce death rates even more. As a result, tracking changes in the burden of MSK disorders over time has become crucial for the creation of health policy.

Recent results from Global Burden of Disease (GBD) research on the effects of MSK disorders have mostly focused on macro-level evaluations, both internationally and locally. Previous studies have assessed worldwide MSK disease trends and associated risks from 1990 to 2021, investigated how they connect to socioeconomic development, and provided projections for the burden of MSK disorders in the future [4,5,7]. However, most of these studies have mostly taken a global approach to the problem, neglecting to look into the variations across various countries and regions and, thus, ignoring the unique situations of certain nations. The challenges posed by MSK disorders have drawn significant attention from the medical profession in China, the most populous country in the world. The effect of MSK disorders in China has been the subject of a few relevant studies, some studies found that musculoskeletal disorders were the third leading cause of Non-communicable diseases (NCDs) DALYs [8,9], the YLD due to musculoskeletal disorder ranked first [10], Sun Yongsheng discovered that in certain regions of our country, the proportion of MSK increases much more rapidly with age compared to other populations [11]. However, it has not yet been thoroughly investigated how MSK disorders have developed in the Chinese population. The burden of MSK disorders in China is therefore thoroughly analyzed and compared to worldwide estimates from 1990 to 2021 using the most recent GBD data. We conducted a thorough study of the evolution of the burden over the past three decades, considering both age and gender perspectives, and we analysis to look at the temporal patterns of MSK disorders. The goal is to give decision-makers the essential knowledge they need to assess the overall burden of MSK disorders in China, which will help them develop focused preventative strategies and allocate public health resources equitably.

## Methods

### Data source

The GBD 2021 dataset is a comprehensive database of incidence, prevalence, and mortality for more than 300 diseases and injuries [12]. To estimate research participants based on variables like age, gender, location, and year, many illnesses and injuries require modeling of processed data using standardized procedures. The Cause of Death Ensemble model (CODEm) and DisMod-MR that is a Bayesian meta-regression tool were the main standardization tools used in this study [4]. It finds different variables that perform the best when assessing predictive validity on out-of-sample data and applies a variety of modeling tools to compare ratios or fractions of causes [13,14].

The Global Health Data Exchange (GHDx) and its related tools (http://ghdx.healthdata. org/gbd-results-tool) provided the data on musculoskeletal (MSK) disorders, including RA, OA, LBP, NP, and other musculoskeletal disorders (OMSKD), used in this study. Detailed methodology for the GBD study is reported elsewhere [4]. In order to assess the effect of MSK disorders, we used the GBD tool to collect data on incidence rates, prevalence rates, mortality rates, and disability-adjusted life years (DALYs) for China and the world between 1990 and 2021.

Using information from the GBD database, we examined the incidence, prevalence, mortality, DALYs, and related age-standardized measures, such as incidence rate (ASIR), prevalence rate (ASPR), mortality rate (ASMR), and DALYs rate (ASDR), for MSK disorders in China and around the world. We also evaluated the crude rates for various age groups, including the crude incidence rate (CIR), crude prevalence rate (CPR), crude mortality rate (CMR), and crude DALYs rate (CDR). We used Joinpoint software from the National Cancer Institute in Rockville, Maryland, USA, to calculate the average annual percentage change (AAPC) and its associated 95% confidence interval (95% CI) in order to assess the trend in disease burden [5,15,16]. A regression model, ln(y)=α + βx + ε, be used to fit the logarithmic age-standardized indicators, where y stands for the corresponding age-standardized indicator and x for the year. The 95% CI also be computed using the model, and the AAPC was determined to be 100 × (exp(β) −1) [17]. The age-standardized indicator exhibits a rising trend if the 95% CI of the related AAPC estimate is more than zero, a falling trend if it is less than zero, and a stable trend if it includes zero.

Statistical analyses were performed with the use of R statistical software, version 4.1.3, and Joinpoint software, version 4.9.1.0. *p* < 0.05 was deemed statistically significant.

## Results

### Description of the burden of MSK disorders in China and worldwide

From 42,000,767 (95% CI: 37,804,103–46,495,427) in 1990 to 66,896,735 (95% CI: 60,375,225–73,192,321) in 2021, there was a cumulative 5.93% rise in the number of MSK disorders cases in China. Like this, the incidence rose 65.31% globally from 1990 to 2021, from 215,339,150 instances (95% CI: 194,696,724–237,685,150) to 355,983,102 cases (95% CI: 322,889,577–389,774,521). Between 1990 and 2021, China’s ASIR dropped from 4039.13 (95% CI: 3648.56–4437.89) per 100,000 to 3634.09 (95% CI: 3310.43–3961.56) per 100,000. As of 2021, the ASIR was 4358.54 (95% CI: 13964.96–4769.78) per 100,000 people worldwide, down from 4641.50 (95% CI: 4195.96–5099.98) per 100,000 in 1990. In contrast, the incidence rate’s AAPC fell by −0.21% (95% CI: −0.22–0.20) worldwide and by −0.34% (95% CI: −0.38–0.30) in China between 1990 and 2021 (Table 1).

**Table 1.** All-age cases and age-standardized incidence, prevalence, mortality, and DALYs rates and corresponding AAPC of MSK in China and globally in 1990 and 2021.

		1990		2021		
		All-ages cases	Age-standardized rates per 100,000 people	All-ages cases	Age-standardized rates per 100,000 people	1990-2019AAPC
Location	Measure	*n*(95%CI)	*n*(95%CI)	*n*(95%CI)	*n*(95%CI)	*n*(95%CI)
China	Incidence	42,000,767 (37,804,103–46,495,427)	4,039 (3,648–4,437)	66,896,735 (60,375,225–73,192,321)	3,634 (3,310–3,961)	−0.34 (−0.38 – −0.30)
	Prevalence	170,594,404 (159,481,066–182,116,267)	16,966 (15,950–17,975)	330,512,567 (311,615,230–349,236,895)	17,358 (16,383–18,394)	0.09 (0.06–0.11)
	Deaths	10,056 (8,354–12,213)	1.22 (1–1.49)	18,572 (14,422–22,318)	1.10 (0.85–1.32)	−0.41 (−0.82 – −0.00)
	DALYs	16,629,496 (12,164,722–22,138,075)	1,615 (1,169–2,151)	29,573,156 (21,119,616–40,348,604)	1,578 (1,138–2,124)	−0.07 (−0.10 – −0.04)
Global	Incidence	215,339,150 (194,696,724–237,685,150)	4,641 (4,195–5,099)	355,983,102 (322,889,577–389,774,521)	4,358 (3,964–4,769)	−0.21 (−0.22 – −0.20)
	Prevalence	865,073,986 (813,102,876–917,749,058)	19,178 (18,084–20,279)	1,629,422,501 (1,546,837,354–1719480265)	19,836 (18,835–20,950)	0.11 (0.09–0.13)
	Deaths	58,380 (52,987–62,644)	1.55 (1.41–1.66)	115,448 (100,173–124,847)	1.47 (1.28–1.59)	−0.25 (−0.41–0.08)
	DALYs	86,285,114 (63,579,335–114,732,382)	1,886 (1,379–2,523)	157,155,505 (114,537,527–210,027,536)	1,916 (1,397–2,558)	0.04 (0.02–0.05)

### Prevalence of MSK disorders in China and worldwide

In 1990, there were 170,594,404 (95% CI: 159,481,066–182,116,267) instances with MSK disorders in China; by 2021, that number had risen to 330,512,567 (95% CI: 3,116,152,30–349,236,895), a cumulative increase of 7.64%. Nonetheless, the frequency rose by 88.36% globally, from 865,073,986 (95% CI: 813,102,876–917,749,058) in 1990 to 1,629,422,501 (95% CI: 1,546,837,354–1,719,480,265) in 2021. As of 2021, the ASPR was 19836.759 (95% CI: 18835.339–20950.312) per 100,000 people, up from 19178.471 (95% CI: 18084.374–20279.512) per 100,000 people in 1990. From 1990 to 2021, China’s ASPR grew from 16966.24 (95% CI: 15950.233–17975.765) per 100,000 people to 17358.703 (95% CI: 16383.82–18394.176). In contrast, the prevalence of AAPC rose by 0.0881% (95% CI: 0.0643–0.1120) in China and by 0.1077% (95% CI: 0.0890–0.1265) globally between 1990 and 2021 (Table 1).

### Deaths of MSK disorders in China and worldwide

In 2021, MSK disorders accounted for 115,448 (95% CI: 100,173–124,847) deaths worldwide, a 97.75% reduction from 1990. Between 1990 and 2021, China’s death rate dropped by 84.69%. Between 1990 and 2021, the ASMR fell from 1.551 (95% CI: 1.41–1.66) per 100,000 people to 1.47 (95% CI: 1.28–1.59) per 100,000 people. In 1990, China’s ASMR was 1.21 (95% CI: 1–1.49) per 100,000 people; by 2021, it had dropped to 1.10 (95% CI: 0.85–1.32) per 100,000 people. In contrast, the AAPC of the mortality rate rose by 1.10% (95% CI: 0.85–1.32) in China and fell by 1.471% (95% CI: 1.28–1.59) globally between 1990 and 2021 (Table 1).

### DALYs of MSK disorders in China and worldwide

Globally, the DALYs for MSK disorders increased by 82.14% from 1990 to 2021, from 86,285,114 (95% CI: 63,579,335–114,732,382) in 1990 to 157,155,505 (95% CI: 114,537,527–210,027,536) in 2021. From 1990 to 2021, China’s DALYs dropped by 77.84%. In 1990, the ASDR was 1886.22 (95% CI: 1379.64–2523.28) per 100,000 people; in 2021, it was 1916.21 (95% CI: 1397.79–2558.49) per 100,000. As of 2021, China’s ASDR was 1578.71 (95% CI: 1138.61–2124.31) per 100,000 people, down from 1615.732 (95% CI: 1169.69–2151.25) per 100,000 in 1990. In contrast, the AAPC of DALYs fell by −0.07 (95% CI: −0.10–0.04) in China and grew by 0.04 (95% CI: 0.02-0.05) globally between 1990 and 2021 (Table 1).

### Joinpoint regression analysis of the burden of MSK disorders in China and worldwide

**Figure 1.**
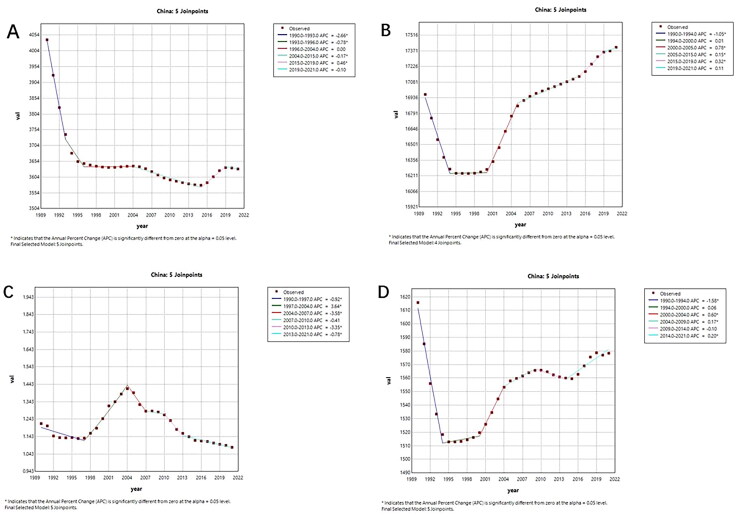
The APC of ASIR, ASPR, ASMR, and ASDR of MSK disorders in China from 1990 to 2021. (a) ASIR, (b) ASPR, (c) ASMR and (d) ASDR. (*means *p*-values < 0.05 and significant results).

**Figure 2.**
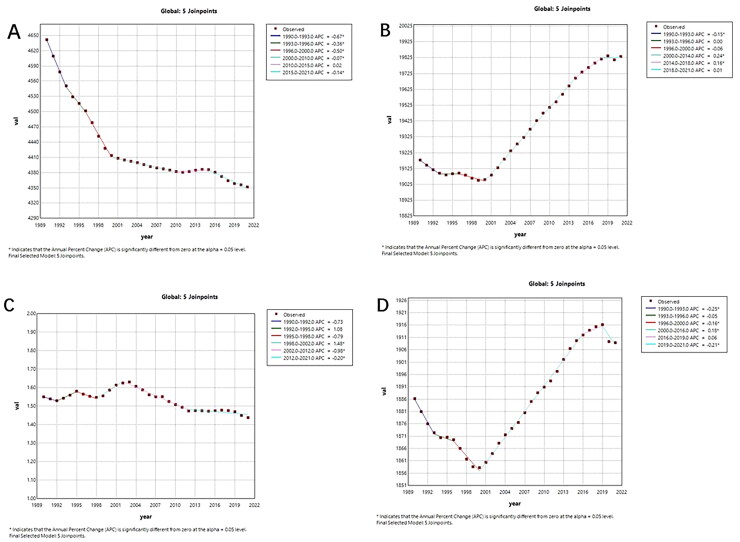
The APC of ASIR, ASPR, ASMR, and ASDR of MSK disorders in global from 1990 to 2021. (a) ASIR, (b) ASPR, (c) ASMR, and (d) ASDR. (*means *p*-values < 0.05 and significant results).

Figures 1 and 2 show the Joinpoint regression analysis of ASIR, ASPR, ASMR, and DALYs for MSK disease in China and globally from 1990 to 2021. The MSK disorders’ annual percentage change (APC) Between 1990 and 1994, ASIR and ASPR in China had notable decreases (ASIR: 1990–1993 APC = −2.66; 1994–1996 APC = −0.78, *p* < 0.05; ASPR: 1990–1994 APC = −1.05, *p* < 0.05). While ASPR started to clearly indicate an increase trend after 2000 (*p* < 0.05), ASIR continued to exhibit a negative trend after 1995. Apart from 2010–2015 (*p* <0.05), ASIR showed a large global decline from 1990 to 2021. In contrast, ASPR started to rise in 2000 and had a notable increase from 2000 to 2018 (ASPR: 2000–2014 APC = 0.16, *p* <0.05; ASPR: 2014–2018 APC = 0.01, *p* <0.05).

### Trends in the burden of MSK disorders disease in China and worldwide

**Figure 3.**
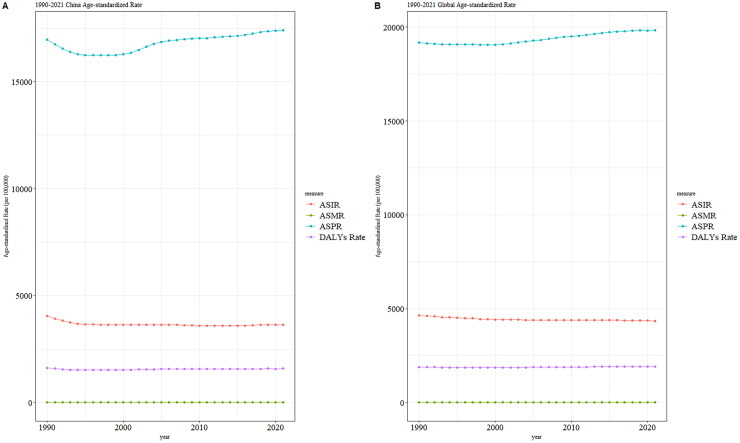
Trend comparison of ASIR, ASPR, ASMR, and ASDR of MSK disorders in China and worldwide from 1990 to 2021.

The ASPR of MSK disorders in China and throughout the world exhibits a consistent and moderate rising trend between 1990 and 2021, as shown in Figure 3. The levels of ASIR, ASMR, and ASDR were general.

### Burden of MSK disorders in different age groups in China in 1990 and 2021

**Figure 4.**
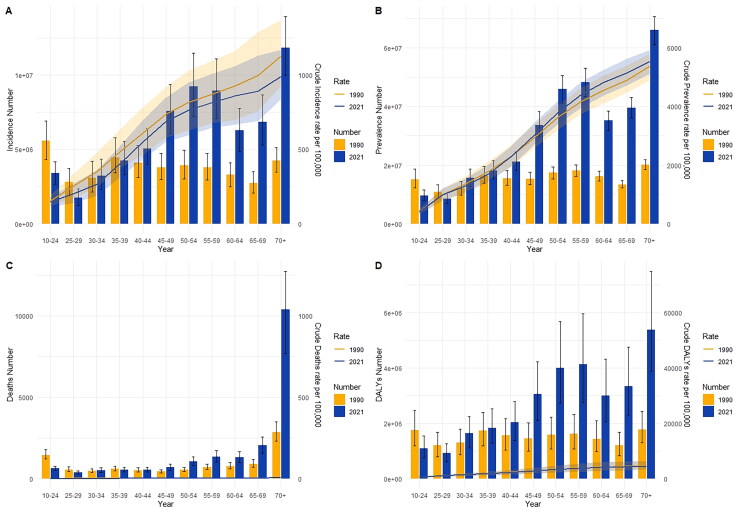
Comparative of the incidence, prevalence, mortality, and DALYs counts, along with their crude rates, by age group in China from 1990 and 2021. (a) Incident cases and CIR; (b) prevalent cases and CPR; (c) death cases and CMR; (d) DALYs counts and CDR; bar charts represent counts; lines represent crude rates.

A comparison of the incidence, prevalence, mortality, and DALYs of MSK disorders in China in 1990 and 2021 across various age groups, as well as the associated crude rates, was shown in Figure 4. According to this finding, the number of MSK disorders cases in China rose sharply after the age of 25 and fell sharply after the age of 60 in 2021, as compared to the fluctuation of MSK disorders cases in all age groups in 1990. The crude incidence rate (CIR) of MSK disorders rose with age in 1990 and 2021. However, compared to China, the worldwide CIR for MSK disorders had a lower incidence rate, albeit showing the same upward trend across all age categories (Figure 4A, Supplementary Figure 1).

CPR showed comparable patterns (Supplementary Figure 1). The age group of 55 to 59 years old had the highest frequency of MSK disorders in China in 1990 and 2021. (Figure 4B). In terms of fatalities, the age group that died the most in 1990 and 2021 was 65–69 years old (Figure 4C). CDR showed similar patterns, rising as people aged. Like the shifting patterns in worldwide CDR, the highest DALYs in 1990 occurred in the 35–39 age group and in 2021 in the 55–59 age group (Figure 4D) (Supplementary Figure 1).

### Gender disparities in the burden of MSK disorders in different age groups in China

**Figure 5.**
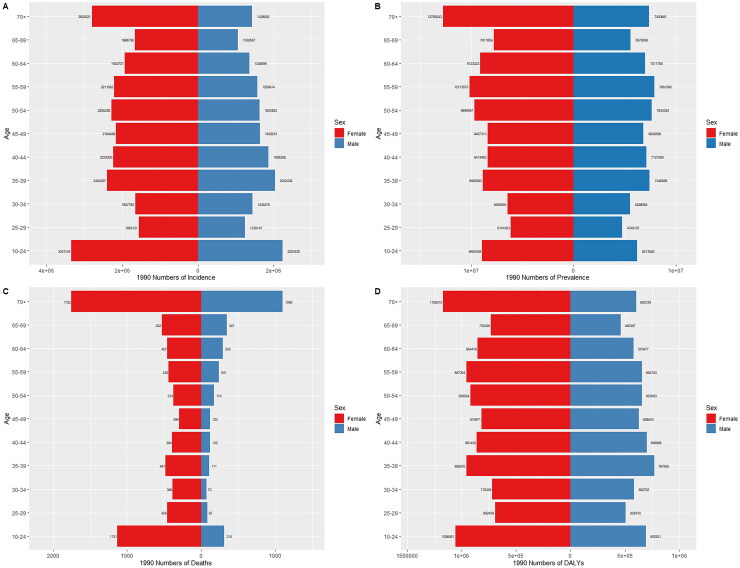
Comparison of the number of incidence, prevalence, mortality, and DALYs of MSK disorders in males and females of different age groups in China in 1990. (a) Incidence; (b) prevalence; (c) mortality; (d) DALYs.

**Figure 6.**
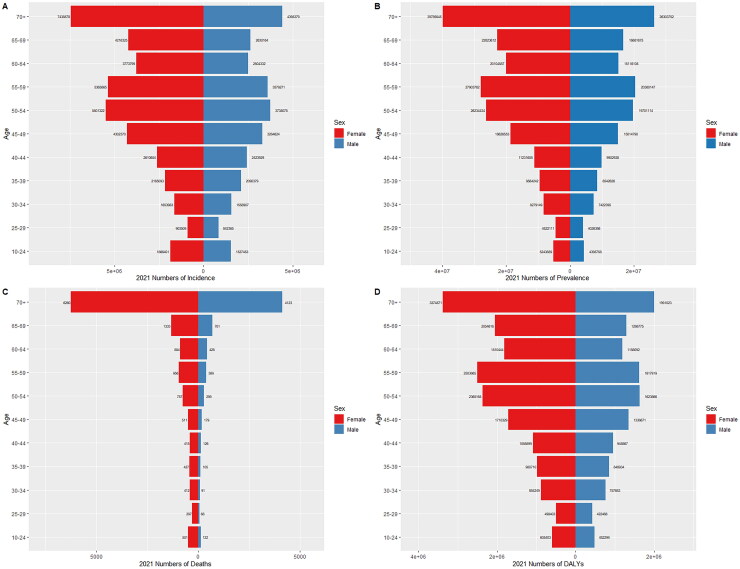
Comparison of the number incidence, prevalence, mortality, and DALYs of MSK disorders in males and females of different age groups in China in 2021. (a) Incidence; (b) prevalence; (c) mortality; (d) DALYs.

The incidence, prevalence, mortality, and DALYs of MSK disorders in China in 1990 and 2021 are shown in Figures 5 and 6 for various age groups of males and females. Based on the incidence data, the 35–39 age group in 1990 had the highest incidence of MSK disorders in both men and females, and the 50–54 age group in 2021 had the highest incidence in both genders. Females were more likely than men to have MSK abnormalities across all age categories in 1990 and 2021 (Figures 5A and 6A).

According to the 1990 prevalence statistics, both men and women with MSK disorders were more prevalent after the age of 35, with the 55–59 age group seeing the biggest prevalence surge. Females were more likely than men to have MSK disorders across all age categories in 1990 and 2021 (Figures 5B and 6B). Globally, the similar pattern has been seen (Supplementary Figure 2). In addition, DALYs and fatalities among males in 1990 were greater among females across all age categories, with the highest number for both genders occurring between the ages of 55 and 59.

The study found that more women died than men in all age groups in 2021, and the number of deaths increased with age, with the highest deaths occurring in those aged 65 to 69 years. Globally, a similar trend exists (Supplementary Figure 2, Figures 5C and 6C). In all age categories, females had more DALYs than men, after the age of 24 years, DALYs gradually increased with increasing age, reaching a peak between the ages of 55 and 59 years, followed by a decline (Figures 5D and 6D).

**Figure 7.**
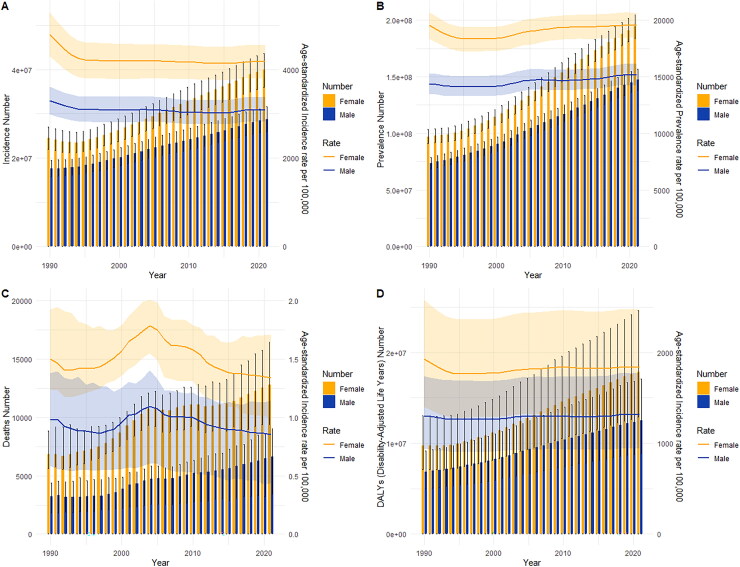
Comparison of full-age cases and age-standardized rates of incidence, prevalence, mortality and DALYs among men and women in China from 1990 to 2021. (A) Incident cases and ASIR; (B) Prevalent cases and ASPR; (C) Deaths cases and ASMR; (D) DALYs counts and ASDR. Bar charts represent counts; lines represent age-standardized rates.

The illness burden and age-standardized rates of MSK disorders in China from 1990 to 2021 were compared for males and females of all ages in Figure 7. The ASIR of MSK disorders in men and females, which peaked in 1990 and showed the biggest gender disparity, is depicted in Figure 7A. Then, as the number of years increased, the ASIR fell. Between 1990 and 2001, the ASPR of MSK disorders in both boys and females steadily declined. From 2002 to 2009, it slightly climbed, but after 2010, it leveled out. Females consistently had a greater ASPR than men (Figure 7B). Similar patterns are happening all throughout the world, which worries us (Supplementary Figure 3).

We note that in 1990 there were large differences in the number of deaths and ASMR for all age groups for both men and women, with ASMR being much higher for women than for men (Figure 7C). In the years after 2010, both the overall ASMR and the gender gap significantly diminished. Results from ASDR of MSK disorders were comparable to those from ASMR, and the worldwide trend was in line with that (Figure 7D, Supplementary Figure 3)

## Discussion

We examined how the illness burden of MSK disorders varied by gender and age in China and worldwide. The findings demonstrated that, across all measures, China and the rest of the globe are on the same development trajectory. Between 1990 and 2021, there was a consistent and slow increase in the ASPR of MSK disorders in China and across the world. Since MSK encompasses a variety of diseases, we conducted a classification study on multiple subtypes, including OA, LBP, RA, and NP. See Supplement Table S1–S4. We found that all the death cases in the MSK group were caused by RA. Some studies found that RA is associated with increased mortality rates compared with the general population in the majority of cohorts published, and the expected survival of RA patients is likely to decrease 3–10 years [18]^.^ Similar changes were observed in ASIR, ASMR, and ASDR. The age of the patients had an impact on the incidence, prevalence, mortality, and DALYs of MSK disorders; MSK disorders were more common in younger people and had higher incidence and death in older people. According to the gender composition, women were more likely than men to get MSK disorders.

According to our study, excluding those aged 10 to 24 years, the 35–39 years old group had the highest incidence, prevalence, mortality and DALYs of MSK disease in 1990 in China and the world, 50–54 in 2021. This might be because adolescence brings about biological, psychological, and social changes that increase the risk of MSK problems in this age group. For instance, musculoskeletal health may be impacted by hormonal changes specific to this developmental period [19]. This might be because adolescence brings about biological, psychological, and social changes that increase the risk of MSK problems in this age group. For instance, musculoskeletal health may be impacted by hormonal changes specific to this developmental period [20]. A substantial decline occurs after age 60, though, maybe because of reaching retirement age and no longer needing to work. Prior research indicates that an uncomfortable work position and strenuous physical labor raise the risk of MSK disorders [7,21]. Ziyi Jin [22] conducted an analysis of age-specific DALY years for MSK globally, regionally and nationally from 1990 to 2019. They discovered that in both established and emerging regions, the relationships between the sociodemographic index and the anticipated yearly percentage change of MSK diseases were inverted. Various age groups exhibit distinct disease-specific patterns. At the worldwide level and in the majority of GBD regions, the ASDR of RA declined dramatically among boys and girls aged 0–14 years, with the most noticeable decline occurring in southern sub-Saharan Africa (SSA). However, it increased somewhat in other age groups. While the ASDR of NP declined in the 0–14 and 15–49 age groups, but seemed steady among the >50 age groups, the ASDR of LBP declined dramatically across sexes and age groups, particularly in the 15–49 age group. On the other hand, gout and other MSK illnesses had the greatest increase in ASDR across all age groups and sexes worldwide, as well as in the majority of GBD locations.

According to the study’s findings, ASIR and ASPR increased both internationally and in China between 1990 and 2021. China may be considered a high-middle-SDI country. Can Chen et al. [23] discovered that while the worldwide years lived with disability (YLDs) rate decreased somewhat between 1990 and 2019 (AAPC = −0.04%, 95% CI: −0.06% to −0.03%), it grew considerably in high, low-middle, and low SDI regions. According to some studies, the number of DALYs instances of MSK disorders in China has been steadily increasing over the last 30 years, highlighting the critical need to prevent and cure all forms of disease. LBP in particular, which makes up the majority of the illness burden associated with MSK problems, suggests that prevention and treatment of this condition need to be a primary focus of China’s public health agenda [8].

Additionally, our analysis verified that, both in China and worldwide, the incidence and prevalence of MSK disorders were greater in women than in males across a range of age groups. Age and gender may also be linked to musculoskeletal problems [24–26]. Yunfa Wang et al. predicted the age-standardized indicators for females will continue to be higher than those for males from 2021 to 2030 [8]. Interestingly, compared to 2019, we found that the incidence of MSK disorders for females aged 30–34 showed a significant decline in 2021, which might be related to changing fertility concepts in China [8,27]. According to data from the Brazilian social security system, musculoskeletal disorders caused a 199% rise in absenteeism between 2004 and 2013, with women seeing a higher increase (216%) than males (187%) [28]. Type I and type II muscle fiber distribution, body composition and structure, and hormone variations might all be biological factors contributing to the disparity in physical performance [29–31]. Because most workstations and instruments are made for males, other research indicate that the increased frequency of musculoskeletal problems may also be explained by the absence of ergonomic adaptations for women [32].

## Conclusion

The incidence, prevalence, mortality, and DALYs of MSK disorders rose in China between 1990 and 2021, demonstrating the growing burden of MSK disorders in that country. Its frequency, morbidity, and mortality are higher in people between the ages of 55 and 60, indicating that its burden varies with age. In addition to having a higher chance of dying, women are more prone than males to acquire MSK disorders. Because of its vast and aging population, MSK disorders continue to be a major public health concern in China.

## Limitation

There were many restrictions on our investigation. Initially, it was an examination of secondary data from the GBD 2021. Therefore, although the results of this study cannot be externally checked, the quality and amount of accessible data greatly influence the accuracy of the results. Second, even among nations with comparable SDI, there are significant differences in health development [4,33].

However, due to a lack of data on the effects of diverse health systems in different nations or areas, we were unable to account for this in the current study. Third, a few other MSK disorder cases were categorized as ‘other MSK disorders’ and were not assessed independently.

## Supplementary Material

Supplemental Material

## Data Availability

The data that support the findings of this study are available from the corresponding author, Haoliang Zhao, upon reasonable request.
