# Supplementary material for: Comparison of the burden of musculoskeletal disorders between China and worldwide data using the global burden of disease dataset from 1990 to 2021
Source: Ann Med. 2025 Jul 13;57(1):2529578. doi: 10.1080/07853890.2025.2529578 (PMC12258175; doi:10.1080/07853890.2025.2529578)
Supplement: Supplemental Material [file IANN_A_2529578_SM6039.zip › suppl_data/Clean copy - Supplementary_Table_1 - IANN-2025-1022.R1.docx]

**Table S1** All-age cases and age-standardized incidence, prevalence, mortality, and DALYs rates and corresponding AAPC of Rheumatoid arthritis (RA) in China and globally in 1990 and 2021

| **Location** | **Measure** | **1990** |  | **2021** |  |
| --- | --- | --- | --- | --- | --- |
|  |  | **All-ages cases** | **Age-standardized rates per 100,000 people** | **All-ages cases** | **Age-standardized rates per 100,000 people** |
|  |  | **n(95%CI)** | **n(95%CI)** | **n(95%CI)** | **n(95%CI)** |
| China | Incidence | 127,826 (111,477-145,914) | 11.59 (10.15-13.15) | 247,307 (216,205-282,998) | 13.70 (12.12-15.55) |
|  | Prevalence | 2,041,682 (1,746,913-2,391,022) | 205.71 (177.56-238.18) | 4,755,487 (4,141,219-5,452,492) | 240.70 (210.77-277.95) |
|  | Deaths | 4,776 (3,903-5,934) | 0.70 (0.57-0.85) | 10,279 (7,411-12,615) | 0.54 (0.39-0.66) |
|  | DALYs | 403,058 (307,641-526,244) | 42.37 (33.04-54.32) | 833,818 (621,520-1,083,523) | 42.20 (31.30-55.45) |
| Global | Incidence | 488,269 (435,015-545,895) | 10.42 (9.32-11.64) | 1,000,319 (902,687-1,114,213) | 11.80 (10.64-13.12) |
|  | Prevalence | 7,959,055 (7,041,419-9,085,469) | 182.54 (161.59-207.48) | 17,924,667 (15,973,178-20,303,303) | 208.90 (186.34-236.33) |
|  | Deaths | 21,671 (19,287-24,202) | 0.61 (0.54-0.68) | 37,330 (31,060-43,136) | 0.45 (0.37-0.52) |
|  | DALYs | 1,545,699 (1,201,479-1,977,786) | 36.42 (28.71-46.00) | 3,075,303 (2,310,381-3,974,046) | 35.90 (26.95-46.46) |
