# Supplementary material for: Comparison of the burden of musculoskeletal disorders between China and worldwide data using the global burden of disease dataset from 1990 to 2021
Source: Ann Med. 2025 Jul 13;57(1):2529578. doi: 10.1080/07853890.2025.2529578 (PMC12258175; doi:10.1080/07853890.2025.2529578)
Supplement: Supplemental Material [file IANN_A_2529578_SM6039.zip › suppl_data/Clean copy - Supplementary_Table_2 - IANN-2025-1022.R1.docx]

**Table S2** All-age cases and age-standardized incidence, prevalence, mortality, and DALYs rates and corresponding AAPC of Low back pain (LBP) in China and globally in 1990 and 2021

| **Location** | **Measure** | **1990** |  | **2021** |  |
| --- | --- | --- | --- | --- | --- |
|  |  | **All-ages cases** | **Age-standardized rates per 1**  **00,000 people** | **All-ages cases** | **Age-standardized rates per 100,000 people** |
|  |  | **n(95%CI)** | **n(95%CI)** | **n(95%CI)** | **n(95%CI)** |
| China | Incidence | 29,843,970 (26,065,824-34,012,369) | 2859.38 (2508.62-3225.53) | 43,374,995 (37,494,376-49,159,184) | 2342.46 (2058.05-2639.32) |
|  | Prevalence | 68,281,007 (59,158,022-77,853,897) | 6635.49 (5770.68-7472.80) | 100,093,746 (87,128,173-113,014,316) | 5342.10 (4660.41-5976.28) |
|  | DALYs | 7,772,958 (5,520,145-10,545,676) | 749.03 (530.01-1013.84) | 11,297,805 (7,931,468-15,328,056) | 603.03 (427.63-810.16) |
| Global | Incidence | 165,063,882 (145,785,269-185,933,884) | 3535.00 (3133.04-3960.99) | 266,873,321 (235,369,489-299,406,380) | 3176.63 (2811.82-3562.29) |
|  | Prevalence | 386,731,361 (341,581,662-434,164,620) | 8391.58 (7381.14-9367.39) | 628,838,475 (551,834,407-700,881,341) | 7463.13 (6575.68-8321.80) |
|  | DALYs | 43,386,226 (31,083,937-58,355,210) | 937.34 (669.13-1261.00) | 70,156,962 (50,194,205-9,4104,688) | 832.18 (595.85-1115.24) |
