# Supplementary material for: Comparison of the burden of musculoskeletal disorders between China and worldwide data using the global burden of disease dataset from 1990 to 2021
Source: Ann Med. 2025 Jul 13;57(1):2529578. doi: 10.1080/07853890.2025.2529578 (PMC12258175; doi:10.1080/07853890.2025.2529578)
Supplement: Supplemental Material [file IANN_A_2529578_SM6039.zip › suppl_data/Clean copy - Supplementary_Table_3 - IANN-2025-1022.R1.docx]

**Table S3** All-age cases and age-standardized incidence, prevalence, mortality, and DALYs rates and corresponding AAPC of Neck pain (NP) in China and globally in 1990 and 2021

| **Location** | **Measure** | **1990** |  | **2021** |  |
| --- | --- | --- | --- | --- | --- |
|  |  | **All-ages cases** | **Age-standardized rates per 1**  **00,000 people** | **All-ages cases** | **Age-standardized rates per 100,000 people** |
|  |  | **n(95%CI)** | **n(95%CI)** | **n(95%CI)** | **n(95%CI)** |
| China | Incidence | 6,192,332 (4,778,922-7,748,853) | 558.53 (441.06-693.01) | 10,292,099 (8,062,751-13,039,595) | 567.23 (448.50-699.80) |
|  | Prevalence | 26,530,055 (20,347,966-32,917,309) | 2479.42 (1942.66-3042.93) | 48,377,404 (37,665,091-60,063,296) | 2549.87 (2007.89-3141.63) |
|  | DALYs | 2,675,172 (1,740,002-3,911,199) | 248.00 (163.93-353.44) | 4,807,593 (3,155,340-6,903,310) | 254.77 (166.90-357.93) |
| Global | Incidence | 24,903,379 (19,628,380-30,674,274) | 513.21 (404.32-630.08) | 43,286,061 (33,941,594-52,883,959) | 519.28 (407.85-633.38) |
|  | Prevalence | 114,601,451 (88,840,738-141,520,155) | 2436.71 (1912.98-2992.64) | 206,029,629 (161,756,683-252,863,254) | 2443.02 (1923.03-3002.33) |
|  | DALYs | 11,442,356 (7,608,943-16,334,313) | 241.96 (162.05-343.53) | 20,415,497 (13,638,705-28,856,643) | 242.30 (162.60-342.76) |
