# Supplementary material for: Comparison of the burden of musculoskeletal disorders between China and worldwide data using the global burden of disease dataset from 1990 to 2021
Source: Ann Med. 2025 Jul 13;57(1):2529578. doi: 10.1080/07853890.2025.2529578 (PMC12258175; doi:10.1080/07853890.2025.2529578)
Supplement: Supplemental Material [file IANN_A_2529578_SM6039.zip › suppl_data/Clean copy - Supplementary_Table_4 - IANN-2025-1022.R1.docx]

**Table S4** All-age cases and age-standardized incidence, prevalence, mortality, and DALYs rates and corresponding AAPC of Osteoarthritis (OA) in China and globally in 1990 and 2021

| **Location** | **Measure** | **1990** |  | **2021** |  |
| --- | --- | --- | --- | --- | --- |
|  |  | **All-ages cases** | **Age-standardized rates per 1**  **00,000 people** | **All-ages cases** | **Age-standardized rates per 100,000 people** |
|  |  | **n(95%CI)** | **n(95%CI)** | **n(95%CI)** | **n(95%CI)** |
| China | Incidence | 4,654,141 (4,075,192-5,212,886) | 487.11 (428.13-543.75) | 11,652,721 (10,207,638-13,107,929) | 554.61 (486.85-619.55) |
|  | Prevalence | 53,352,515 (46,603,087-59,685,781) | 6148.92 (5417.29-6855.85) | 152,848,106 (134,655,962-170,842,263) | 7030.67 (6211.20-7831.70) |
|  | DALYs | 1,829,416 (880,108-3,682,520) | 210.61 (101.91-423.86) | 5,327,390 (2,541,781-10,678,675) | 244.79 (117.30-491.91) |
| Global | Incidence | 20,900,510 (18,467,653-23,104,316) | 489.78 (433.10-541.52) | 46,632,144 (41,122,053-51,644,431) | 535.00 (472.38-591.97) |
|  | Prevalence | 256,076,700 (227,119,748-283,438,465) | 6393.12 (5683.20-7059.53) | 606,989,319 (537,873,608-670,519,617) | 6967.29 (6180.70-7686.06) |
|  | DALYs | 8,918,857 (4,264,151-17,983,776) | 222.802 (106.65-450.30) | 21,304,566 (10,189,161-42,935,420) | 244.50 (117.06-493.11) |
