# Supplementary material for: Comparison of the burden of musculoskeletal disorders between China and worldwide data using the global burden of disease dataset from 1990 to 2021
Source: Ann Med. 2025 Jul 13;57(1):2529578. doi: 10.1080/07853890.2025.2529578 (PMC12258175; doi:10.1080/07853890.2025.2529578)
Supplement: Supplemental Material [file IANN_A_2529578_SM6039.zip › suppl_data/Supplementary Figure caption.docx]

**Supplementary Figure 1 (A-D)** Comparative chart of global incidence, prevalence, mortality, DALYs and their age-standardized rates in various age groups between 1990 and 2021

**Supplementary Figure 2 (A-D)** Comparison of the number of incidence, prevalence, mortality, and DALYs of MSK disorders in males and females of different age groups in global in 1990. (A) Incidence; (B) Prevalence; (B) Mortality; (D) DALYs

**Supplementary Figure 3 (E-H)** Comparison of the number of incidence, prevalence, mortality, and DALYs of MSK disorders in males and females of different age groups in global in 2021. (E) Incidence; (F) Prevalence; (G) Mortality; (H) DALYs

**Supplementary 4 (A-D)** Comparison of full-age cases and age-standardized rates of incidence, prevalence, mortality and DALYs among men and women in global from 1990 to 2021. (A) Incident cases and ASIR; (B) Prevalent cases and ASPR; (C) Deaths cases and ASMR; (D) DALYs counts and ASDR. Bar charts represent counts; lines represent age-standardized rates
