# Supplementary figures and images for: Comparison of the burden of musculoskeletal disorders between China and worldwide data using the global burden of disease dataset from 1990 to 2021
Source: Ann Med. 2025 Jul 13;57(1):2529578. doi: 10.1080/07853890.2025.2529578 (PMC12258175; doi:10.1080/07853890.2025.2529578)

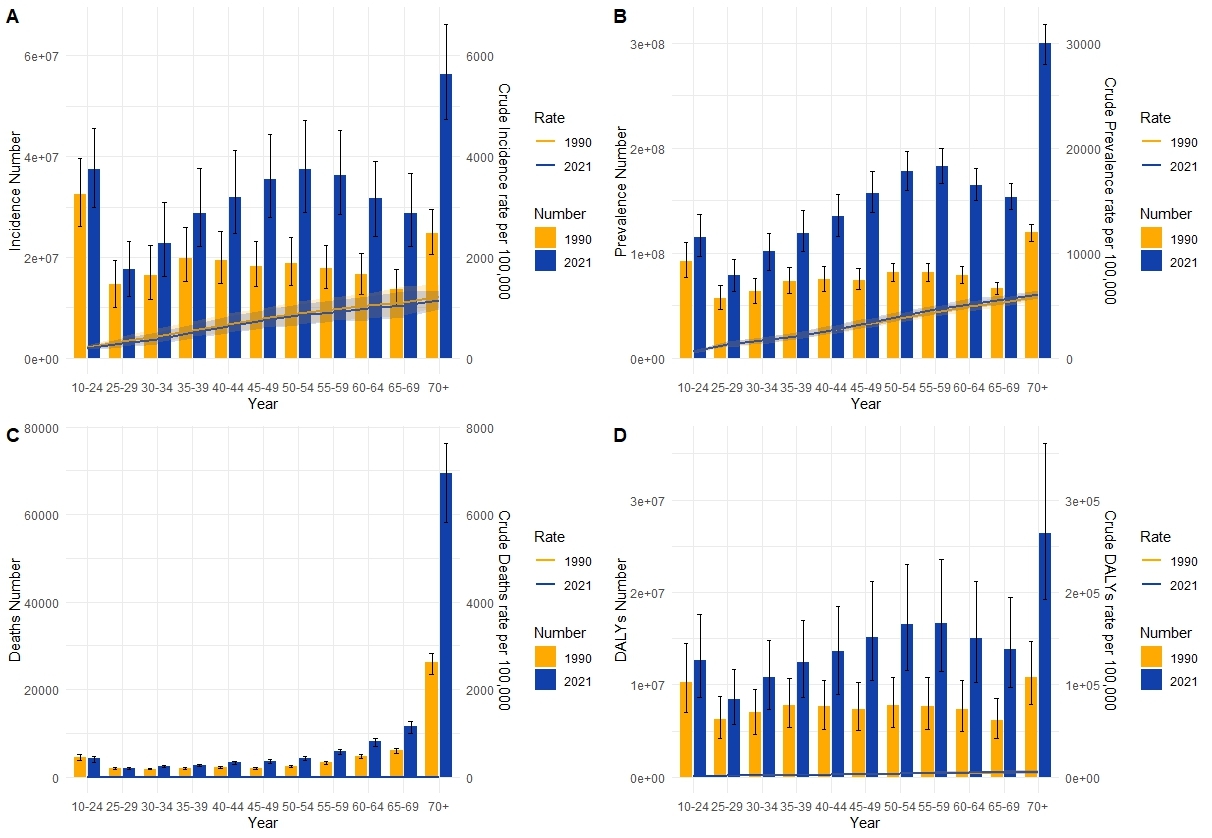

Supplement: Supplemental Material [file IANN_A_2529578_SM6039.zip › suppl_data/Supplementary Figure 1.jpeg]

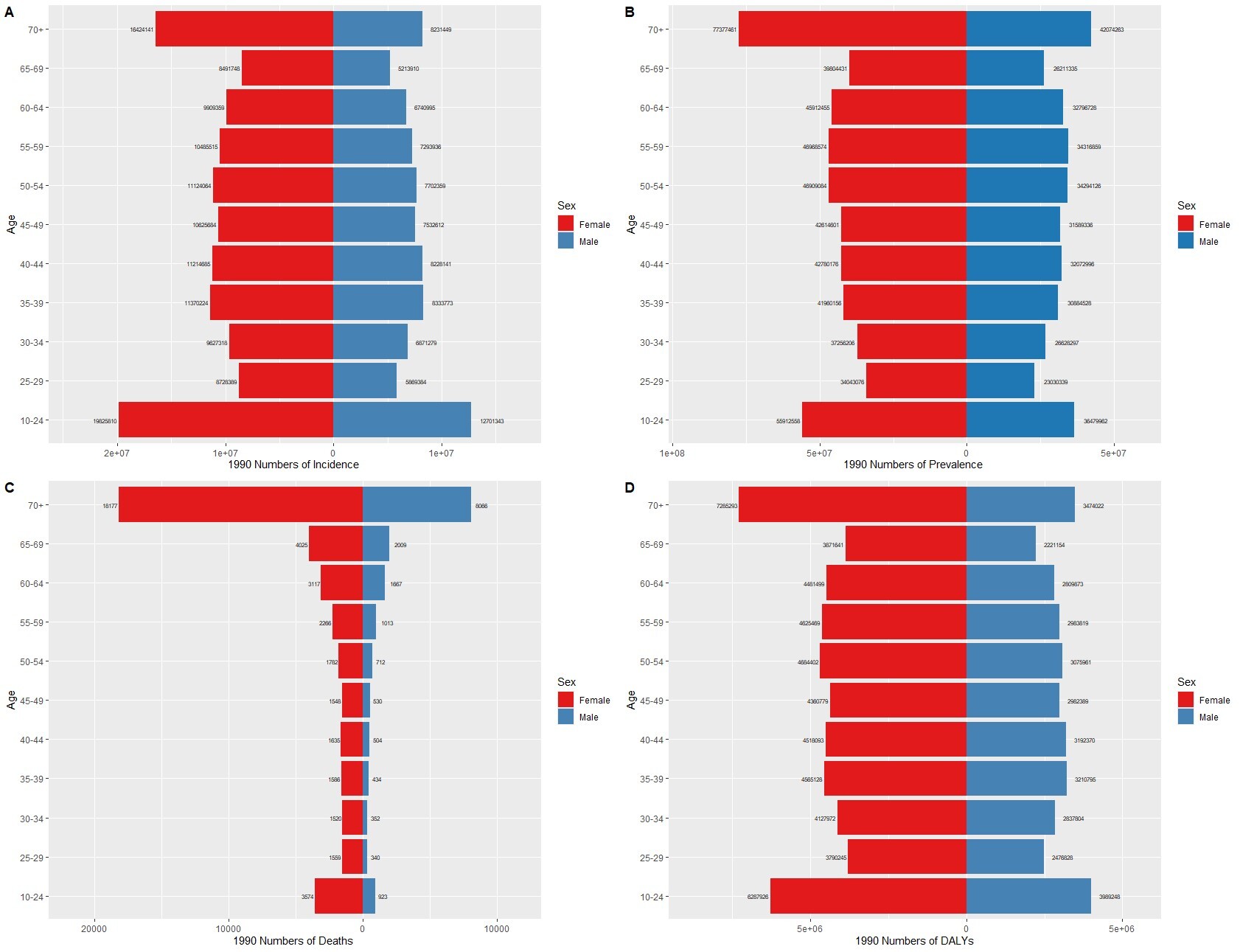

Supplement: Supplemental Material [file IANN_A_2529578_SM6039.zip › suppl_data/Supplementary Figure 2.jpg]

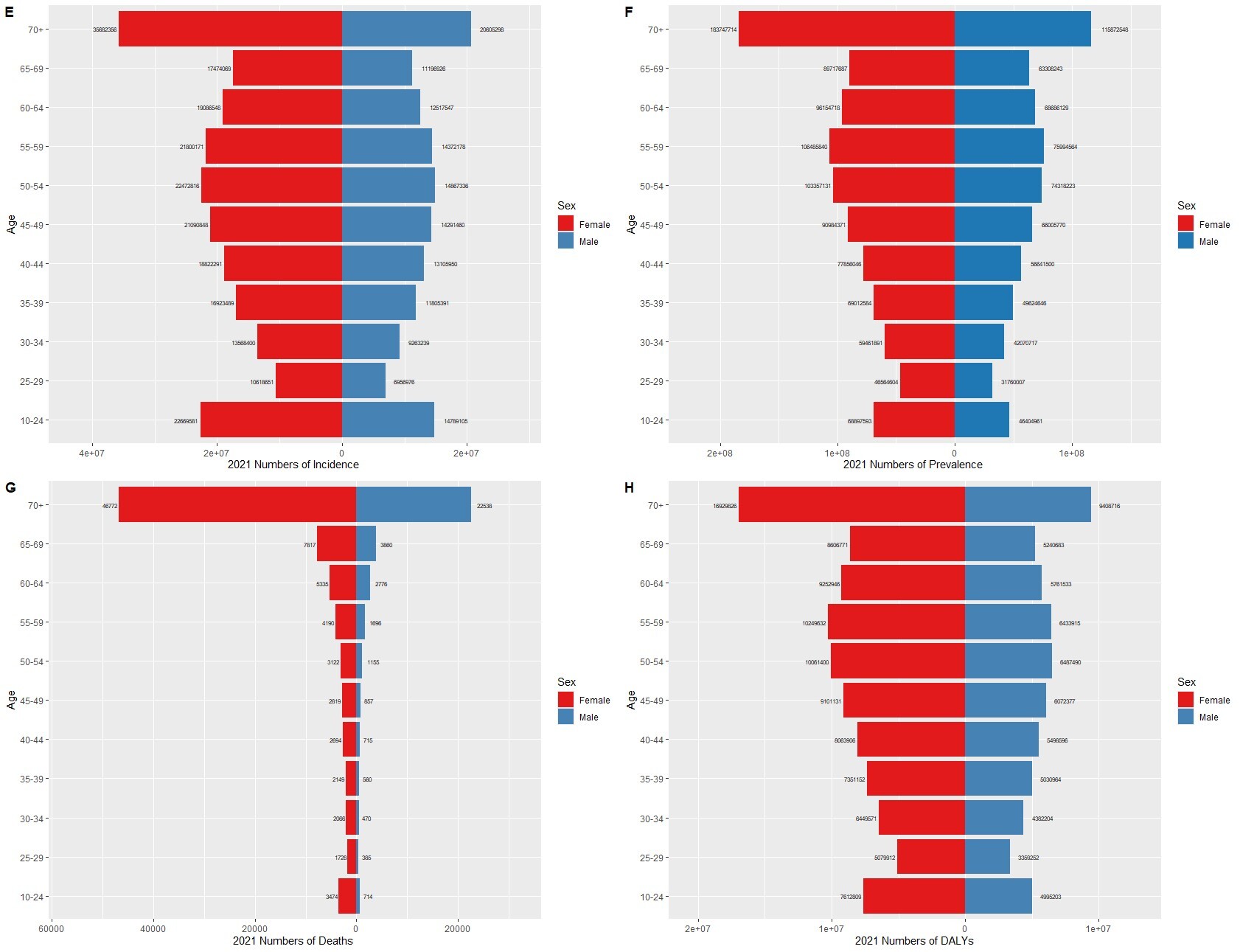

Supplement: Supplemental Material [file IANN_A_2529578_SM6039.zip › suppl_data/Supplementary Figure 3.jpg]

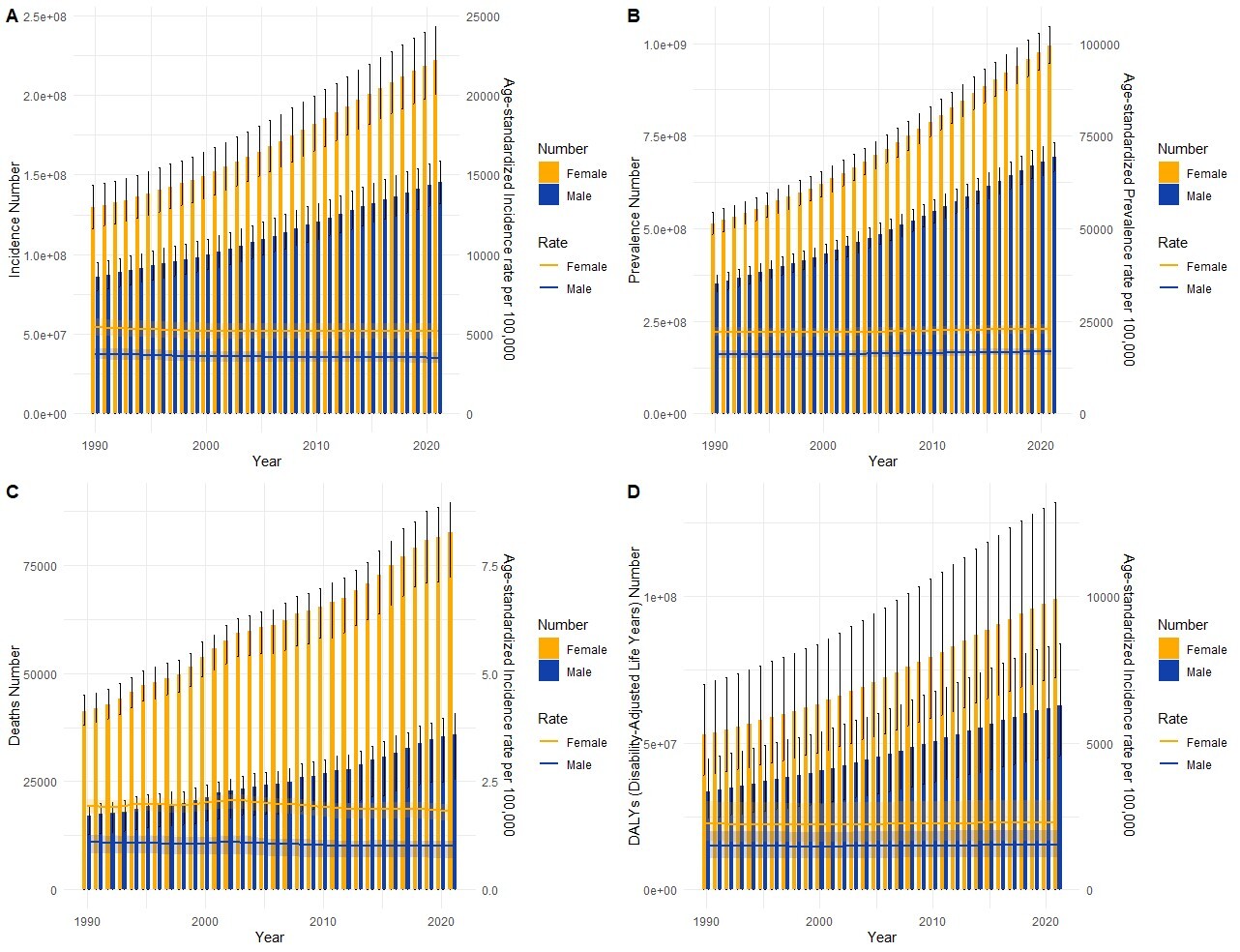

Supplement: Supplemental Material [file IANN_A_2529578_SM6039.zip › suppl_data/Supplementary Figure 4.jpg]
